# Enhanced performance of proton exchange membrane fuel cells by Pt/carbon/antimony-doped tin dioxide triple-junction catalyst

**DOI:** 10.1038/s41598-023-50080-w

**Published:** 2023-12-27

**Authors:** Du-Cheng Tsai, Bing-Hau Kuo, Hung-Pin Chen, Erh-Chiang Chen, Fuh-Sheng Shieu

**Affiliations:** Department of Materials Science and Engineering, National Chung Hsing University, Taichung, 40227 Taiwan, ROC

**Keywords:** Energy science and technology, Materials science

## Abstract

A composite material comprising carbon black and Sb-doped SnO_2_ (ATO) is employed as a support for a Pt catalyst in a membrane electrode assembly (MEA) to improve the performance of a proton-exchange membrane fuel cell under low-humidity conditions. The effects of Sb-doping on the crystal, structural, and electrochemical characteristics of ATO particles are being examined. In a single cell test, the ratio of Sb in ATO is systematically optimized to improve performance. The distribution of Pt nanoparticles is uniform on carbon black and ATO carrier, forming notable triple-junction points at the interface of carbon black and ATO carrier. This structure thus induces a strong interaction between Pt and ATO, promoting the content of metallic Pt. Compared with a Pt/C catalyst, the best-performing Pt/C–ATO catalyst exhibits superior electrochemical activity, stability, and CO tolerance. The power density of MEA with the Pt/C–ATO catalyst is 15% higher than that of the MEA with the Pt/C catalyst

## Introduction

Proton exchange membrane fuel cells (PEMFCs) have emerged as highly promising energy sources due to their exceptional fuel utilization efficiency, low operational temperature, and environmental friendliness^1–3^. Nevertheless, the commercialization of PEMFCs in industrial machinery and electric vehicles continues to face significant challenges regarding cost and reliability^4^. Pt catalysts supported on carbon black (Pt/C) are widely used as primary catalyst layers in PEMFCs. However, the catalytic activity of the Pt/C layer is vulnerable to degradation resulting from CO poisoning and carbon corrosion. In a conventional method of hydrogen production, hydrocarbon reforming is commonly employed because of its cost-effectiveness and high production rate. However, even after undergoing reforming, the resulting fuel may contain residual quantities of CO or comparable CO-like species at the parts-per-million (ppm) level^5^. Notably, CO at the ppm level has marked tendency to be adsorbed onto a Pt surface through a concerted electron transfer mechanism involving the transfer of electrons from the Pt d orbital to the C–O 2π* orbital and simultaneously from the CO 5σ orbital to the Pt orbital^6^. Previous research has demonstrated that even a mere concentration of 10 ppm of CO in a fuel gas can significantly diminish the catalytic activity of Pt catalysts in hydrogen oxidation and reduction reactions^7–9^. Pt catalysts accelerate the corrosion of carbon support materials in PEMFCs^10^. Carbon black, known for its large surface area, high electrical conductivity, and low cost, is commonly utilized to disperse Pt nanocatalysts in PEMFCs. However, when employed as an electrode in PEMFCs, carbon black is exposed to corrosive conditions, such as high humidity, low pH, high potential (0.6–1.2 V), and elevated temperature (~ 80 °C). Under acidic and oxidative environments, carbon corrosion occurs as a thermodynamically plausible process, leading to the loss of carbon supports through their involvement in oxidation reactions during electrochemical processs, as depicted below^11,12^:

$$ {\text{C}} + {\text{2H}}_{{2}} {\text{O}} \to {\text{CO}}_{{2}} + {\text{4H}}^{ + } + {\text{4e}}^{ - } {\text{E}} = 0.{2}0{7}\;{\text{V}}\;{\text{vs}}.\;{\text{RHE}}\;{\text{at}}\;{25}\;^\circ {\text{C}} $$

Borup et al.^13^ demonstrated that carbon corrosion and Pt particle size within a catalyst layer increase with rising potential and decreasing humidity. Wang et al.^14^ investigated performance degradation attributed to Pt loss from a corroded carbon surface and enlargement of Pt particle size within a cathode catalyst layer. To address challenges posed by CO poisoning and carbon corrosion, considerable attention has been directed toward metal oxides. These materials offer high corrosion resistance, exhibit strong interactions with Pt catalysts that enhance activity, and can adsorb abundant hydroxyl groups, facilitating the removal of adsorbed CO-like intermediate species^15–17^.

TiO_2_^18,19^, Nb_2_O_5_^20^, and SnO_2_^21^ metal oxides are widely investigated as catalyst supports owing to their relatively high electrical conductivity, specific surface areas, and chemical stability. Among these metal oxides, SnO_2_ stands out as an n-type semiconductor possessing superior electronic conductivity and cost-effectiveness. Pt–Sn-based catalysts incorporated with Sn demonstrates remarkable catalytic performance attributed to the ability of Sn to decrease Pt binding energy toward the negative side^22^. In addition, SnO_2_ exhibits a co-catalytic effect through surface hydroxyls, as shown in the following formula^23,24^:

$$ {\text{SnO}}_{{2}} + {\text{H}}_{{2}} {\text{O}} \to {\text{SnO}}_{{2}} {-}{\text{OH}}_{{{\text{ad}}}} + {\text{H}}^{ + } + {\text{e}}^{ - } $$

$$ {\text{Pt}}{-}{\text{CO}}_{{{\text{ad}}}} + {\text{SnO}}_{{2}} {-}{\text{OH}}_{{{\text{ad}}}} \to {\text{Pt}} + {\text{SnO}}_{{2}} + {\text{CO}}_{{2}} + {\text{H}}^{ + } + {\text{e}}^{ - } $$

The aforementioned reaction suggests that the incorporation of SnO_2_ into a Pt catalyst facilitates CO oxidation, consequently improving the CO tolerance of Pt. However, the relatively low electrical conductivity and surface area of metal oxides compared with carbon hinders efficient electron transfer during electrochemical reactions. To address this limitation, combining metal oxides and carbon composite materials emerges as a promising approach for modifying electrical conductivity^25–29^. González et al.^30^ synthesized a composite catalyst consisting of Pt–SnO_2_/C using a microwave-assisted polyol method. This composite catalyst demonstrated improved catalytic activity and stability for in methanol oxidation reaction, outperforming commercial Pt–Ru/C catalysts. Another method for improving the conductivity of SnO_2_ is increasing the concentration of free electrons in the SnO_2_ lattice by doping it with metal ions that possess a higher valence state than the native cations in SnO_2_. This type of doping is known as cation doping. Lee et al.^31^ prepared the Pt/Sb-doped SnO_2_ (ATO) catalyst via the polyol method. This catalyst exhibits significantly enhanced oxygen reduction reaction (ORR) activity and CO tolerance compared with Pt/C catalysts.

Current investigations have yet to yield a consensus on the precise Sb content that would constitute the most effective formulation for Pt–SnO_2_/C electrocatalysts. This lack of consensus arises from the fact that the catalytic performance is profoundly reliant on an array of physicochemical traits of the synthesized Pt nanoparticles. To minimize the impact of ATO synthesis on Pt production, this study commenced with the synthesis of ATO/C nanocomposites, followed by the deposition of Pt nanoparticles onto the surface of the ATO/C. Different levels of Sb loading were incorporated to enhance the electrical conductivity of ATO and explore the optimal Sb doping concentration. Our homemade Pt/C results were employed as a reference for comparison with the findings discussed in this paper. We aimed to investigate the impact of Sb doping on the electrical conductivity of SnO_2_ and the dispersion of Pt supported on ATO and carbon composite materials. On the other hand, the activity and performance of many related catalytic materials are evaluated by inducing the ORR in a sulfuric acid solution. Although ORR testing can screen and optimize catalysts at a preliminary stage, it cannot completely replace actual fuel cell tests. The testing in a fuel cell setup is crucial to fully evaluate the potential of a catalyst for practical applications. Therefore, we conducted an evaluation of their performance and stability in single-cell applications. The findings can enhance understanding of the conductivity and electrocatalytic properties of ATO nanoparticles and provide a foundation for the development of high-performance SnO_2_-based materials for fuel cells.

## Experimental

### Catalyst preparation

The ATO/carbon black (C–ATO) particles were synthesized as follows: first, mixtures containing 1 g of carbon black (Vulcan XC-72, Cabot); 0.5 g of SnCl_2_‧2H_2_O (ALFA); and 0.025, 0.05, or 0.1 g of SbCl_3_ (ALFA) dissolved in hydrochloric acid were prepared. The mixtures were then labeled as C–ATO 5%, C–ATO 10%, and C–ATO 20%. These labels are based on the weight of Sb relative to the weight of Sn. The resulting mixtures were thoroughly dispersed by stirring in de-ionized water at 65 °C for 2 h and then treated with ammonia water for the precipitation of hydroxides onto the carbon black support until the pH reached 8.5. The resulting materials were subsequently filtered and dried in a vacuum oven at 105 °C for 12 h to produce C–ATO particles. The method used to prepare ATO particles was the same as that described earlier, but carbon black was not incorporated in the process. The Pt/C–ATO nanoparticles were synthesized using the impregnation method. Approximately 1 g of C–ATO nanoparticle was first added to deionized water and ultrasonicated for 2 h to obtain a good dispersion. Next, 0.66 g of H_2_PtCl_6_‧6H_2_O (Alfa Aesar), 10 mL of 1 M NaOH, and 10 mL of ethylene glycol (ALFA) were added to the solution, which was then refluxed in a three-necked flask at 85 °C for 4.5 h. Finally, the as-prepared Pt/C–ATO catalyst was filtered and dried in a vacuum oven at 85 °C for 12 h. A catalyst ink was prepared from the catalyst, Nafion solution (Dupont), and alcohol (Aldrich), which were mixed using an ultrasonicator for 2 h. The catalyst ink was then sprayed onto commercial carbon cloth (SGL-10BA, SIGRACET). The Pt/C–ATO catalysts were used on the cathode and anode. The Nafion 112 membrane was sandwiched between the anode and cathode and then hot-pressed under 85 kgf cm^−2^ for 2 min at 105 °C. Pt loading at the anode and cathode was 0.4 mg cm^−2^, and the effective area of membrane electrode assembly (MEA) was 5 cm^2^.

### Characterization

The crystal structures were examined using an X-ray diffractometer (XRD, MacScience MXP3) equipped with Cu Kα radiation. The average grain size was determined using Scherrer's formula based on the full width at half-maximum. The morphology analysis was conducted using field emission scanning electron microscopy (SEM, JEOL JSM-6700F). Microstructural characterizations were conducted utilizing an analytical transmission electron microscope (TEM, JEM-2100F). The surface composition and chemical binding energies were investigated employing X-ray photoelectron spectroscopy (XPS, PHI 500 VersaProbe, ULVAC-PHI) equipped with monochromatic Al Kα radiation. The electrical conductivities were determined using the Van der Pauw configuration. The cyclic voltammetry was performed using an electrochemical workstation (CHI 614B) to evaluate the electrochemically active surface area (ECSA) and methanol oxidation reaction (MOR) of the catalysts. The ECSA measurement utilized a 0.5 M H_2_SO_4_ electrolyte with a scan rate of 10 mV/s and a scan range of − 0.4 to 1.2 V, while the MOR test employed a 0.5 M H_2_SO_4_ + 1 M CH_3_OH electrolyte with the same scan rate and a scan range of 0 to 1.4 V. Ag/AgCl electrode served as the reference electrode, while Pt wires were employed as the counter electrode. The polarization curves of a single cell with a serpentine flow pattern were measured at a potential sweeping rate of 0.5 V min^−1^ at room temperature using a fuel-cell test station (Beam 100 from Beam Associate Co., Ltd.). Hydrogen and oxygen were supplied to both the anode and cathode at a flow rate of 100 SCCM.

## Results and discussion

**Figure 1:**
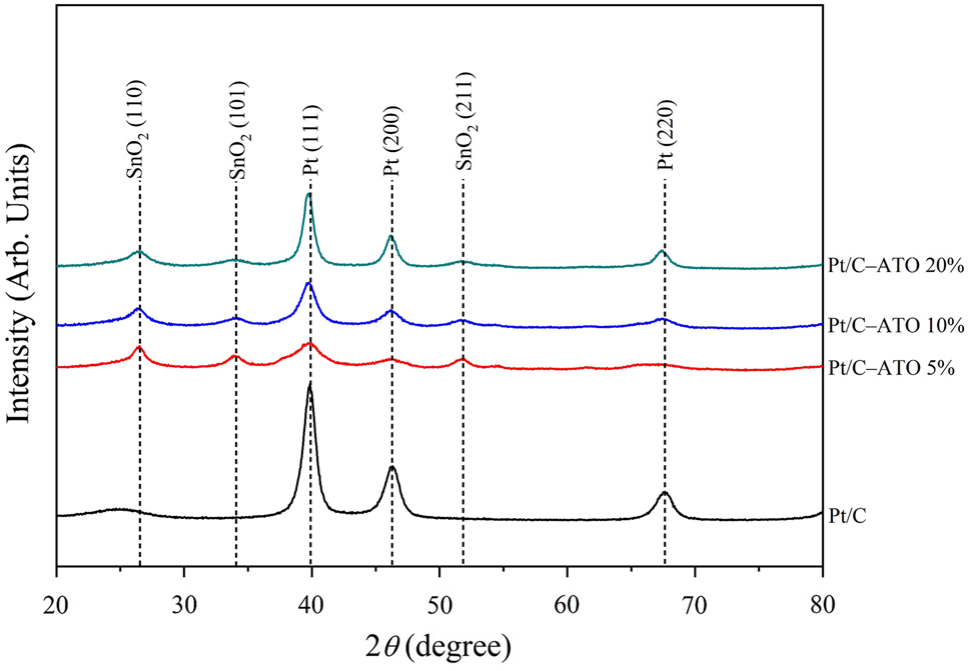
XRD patterns of Pt/C and Pt/C–ATO catalysts.

Figure 1 illustrates the wide-angle diffraction patterns of Pt/C and Pt/C–ATO catalysts. The diffraction peaks corresponding to the crystalline planes of Pt at (111), (200), and (220) were observed at 2θ values of 39.85, 46.21, and 67.75, respectively, as compared with the JCPDS data file (No. 04-0802). Similarly, the diffraction peaks at 2θ values of 26.61, 33.89, 37.95, 38.97, and 51.78 can be assigned to the (110), (101), (200), and (211) planes of SnO_2_ according to the JCPDS data file (No. 00-0411445). The absence of distinct diffraction peaks attributed to antimony oxide in the XRD pattern indicates the incorporation of Sb ions into the SnO_2_ lattice or that antimony oxide is amorphous^31^. To estimate the crystalline sizes of Pt and ATO, Scherrer's equation^32^ was employed, and the results are presented in Table 1. The following phenomena were observed: (1) increase in Sb doping resulted in a notable decrease in the grain size of the SnO_2_ phase (from 4.77 to 3.45 nm) probably because of the incorporation of Sb atoms, which introduce lattice defects and induce internal stress in the crystal lattice^33^. Moreover, the presence of amorphous antimony oxide may have hindered the growth of SnO_2_ crystals. (2) The addition of metal oxides had a certain impact on the size of the Pt particles. However, existing literature does not provide a clear answer regarding how the addition of metal oxides affects the sizes of Pt particles. In some cases, metal oxides can act as stabilizers or promoters for Pt nanoparticles. Metal oxide can interact with Pt nanoparticles through chemical bonding or electrostatic interactions, preventing agglomeration or coalescence and maintaining small particle sizes^27,34–36^. However, metal oxides have lower surface areas than carbon black. This feature may promote particle aggregation and Pt growth, increasing particle size^37,38^. Additionally, when an excess of metal oxides is added, the oxides tend to self-organize into larger clusters, which can lead to the clustering of Pt particles within the interstices of oxide aggregates, consequently decreasing the available surface area for the Pt particles. In the present study, the grain size of Pt is significantly reduced from 4.92 to 2.39 nm after the introduction of ATO. Afterward, the grain size of Pt increased to 6.78 nm with increasing Sb doping. The former may have been dominated by the strong interaction between Pt and ATO, and the latter may have been associated with the reduction in surface area and enhanced agglomeration of ATO at the amount of doped Sb increased.

**Table 1:** The grain size of Pt/C and various Pt/C–ATO catalysts.

Catalyst	Pt grain size (nm)	ATO grain size (nm)
Pt/C	4.92	–
Pt/C–ATO 5%	2.39	4.77
Pt/C–ATO 10%	4.21	3.75
Pt/C–ATO 20%	6.78	3.45

**Figure 2:**
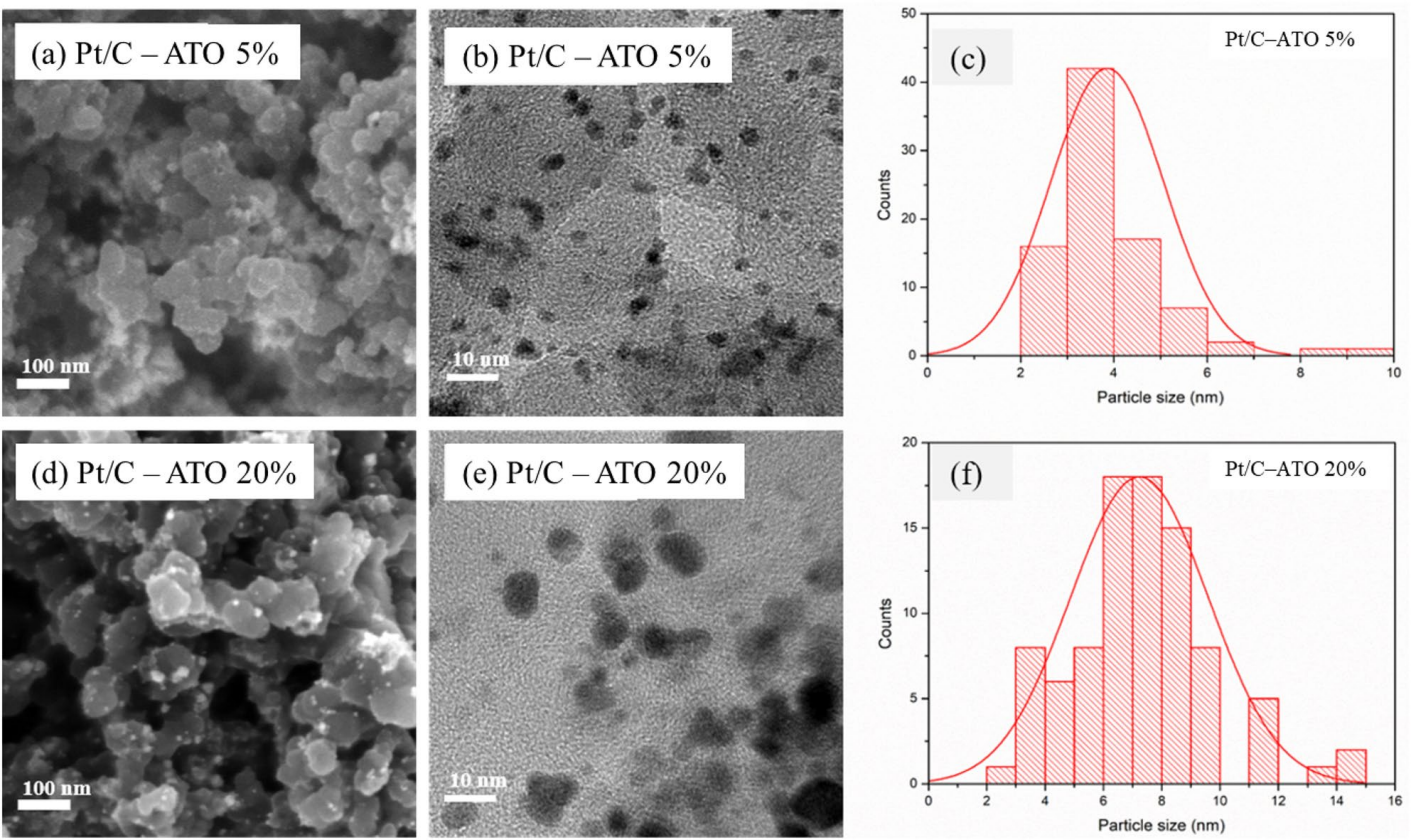
SEM, TEM images, and size distributions of (**a**–**c**) Pt/C–ATO 5% and (**d**–**f**) Pt/C–ATO 20% catalysts.

The FE-SEM images in Fig. 2a and d depict the Pt/C–ATO 5% and 20% catalysts, respectively. These images provide visual evidence of the uniform distribution of Pt and ATO nanoparticles on the carbon black support. Notably, the Pt/C–ATO 20% exhibits visible particle agglomeration, signifying distinctive features associated with this specific composition. The TEM images and corresponding particle size distribution histograms of the Pt/C–ATO 5% and 20% samples are presented in Fig. 2b,c and Fig. 2e,f, respectively. The Pt and ATO nanoparticles in Pt/C–ATO 5% sample exhibited excellent dispersion on the supports, accompanied by a narrow particle size distribution. Additionally, a certain extent of stacking configuration was observed between the Pt and ATO nanoparticles. The average particle size of Pt was determined by directly measuring 100 isolated particles, which yielded an estimated value of approximately 3.86 nm. Although the majority of the Pt and ATO nanoparticles in Pt/C–ATO 20% maintained uniform dispersion on the carbon support, evidence of agglomeration was obtained from a subset of larger particles. The average particle size of Pt increased, reaching a value of 7.23 nm. At an optimal doping level, the dispersion of ATO particles on the surface of carbon black enhances surface heterogeneity and provides additional anchoring points, facilitating the formation of smaller Pt particles. However, when the concentration of ATO particles becomes excessive, their interparticle interactions may promote the formation of larger agglomerates, leading to the localized accumulation and clustering of Pt particles in these regions. The TEM observations are in agreement with the aforementioned SEM and XRD findings.

**Figure 3:**
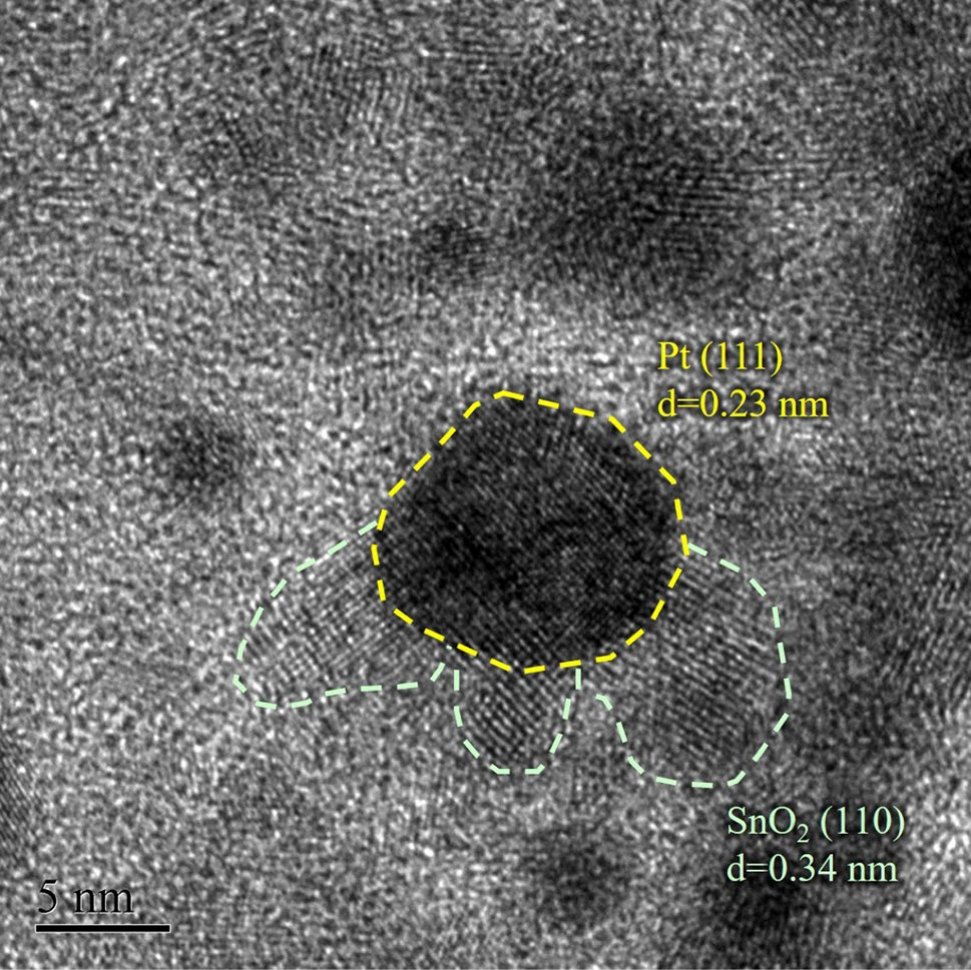
HR-TEM image of Pt/C–ATO 20% catalyst.

The high-resolution TEM image of the Pt/C–ATO 20% catalyst is presented in Fig. 3. Two distinct lattice fringes measuring 0.24 and 0.34 nm were observed, indicating the presence of crystal planes corresponding to Pt (111) and SnO_2_ (110), respectively. The ATO supports effectively served as anchors for the Pt nanoparticles. Specifically, the Pt particles were primarily localized at the boundaries between ATO and carbon black. The emergence of this special triple-junction structure can have a crucial impact that can enhance the stability and activity of Pt catalysts for MORs^39^.

**Figure 4:**
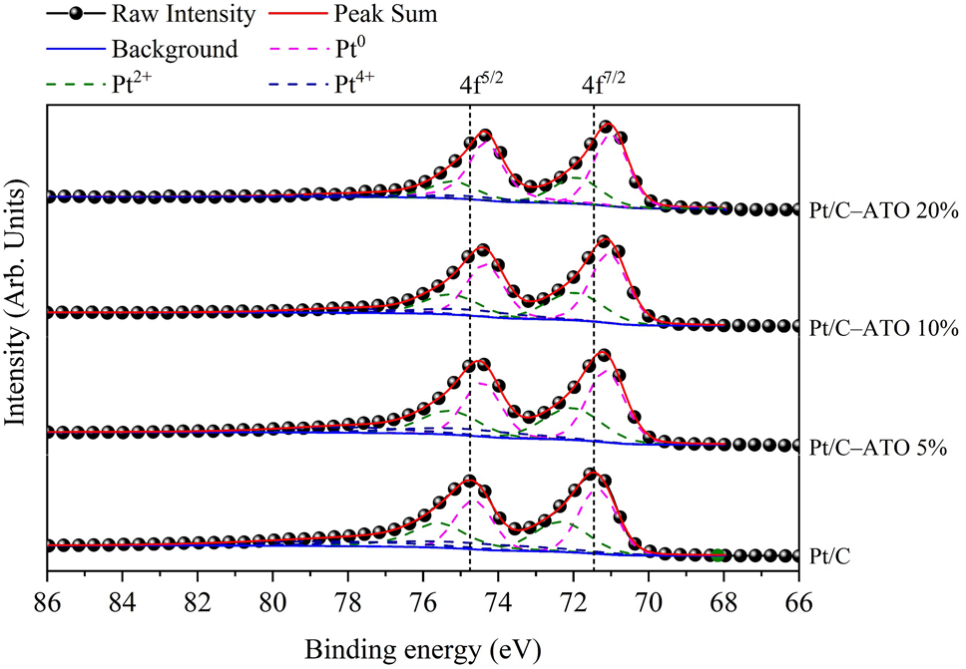
XPS Pt 4f spectra of Pt/C and Pt/C–ATO catalysts.

To shed light on the underlying mechanism behind the promotional role of C–ATO composite supports, XPS was employed to analyze the electronic properties of the Pt catalysts investigated in this study. The high-resolution XPS analysis presented in Fig. 4 involved the deconvolution of the Pt 4f. signals in various catalysts, revealing three distinct components assigned to Pt^0^, Pt^2+^, and Pt^4+^ representing metallic Pt, Pt(OH)_2_, and PtO_2_ like species, respectively^27^. The binding energies of Pt 4f^7/2^, in conjunction with the relative intensity ratio of Pt for each catalyst, are summarized in Table 2. The binding energy values of Pt 4f^7/2^, in conjunction with the relative intensity ratio of Pt for each catalyst, are summarized in Table 2. The binding energy values of all peaks were meticulously aligned with the C 1 s peak position at 284.6 eV, serving as a reference point. The binding energy of metallic Pt in Pt/C–ATO is negatively shifted compared to Pt/C, providing strong evidence for the occurrence of electron transfer from ATO to Pt at Pt/C–ATO triple-junction nanostructures^37^. This effect elevates the Fermi level or reduces Pt d-vacancy within a valence bond (5d orbital)^40,41^.

**Table 2:** The binding energy (eV) and related composition ratio (%) from deconvolution of XPS spectra for Pt 4f region of Pt/C and various Pt/C–ATO catalysts.

Catalyst	Pt^0^		Pt^2+^		Pt^4+^	
	BE (eV)	Ratio (%)	BE (eV)	Ratio (%)	BE (eV)	Ratio (%)
Pt/C	71.36	43.61	72.33	35.54	75.16	20.85
Pt/C–ATO 5%	71.14	49.28	72.03	33.61	74.90	17.11
Pt/C–ATO 10%	71.05	56.02	71.97	31.22	74.79	12.76
Pt/C–ATO 20%	70.10	64.84	71.92	28.34	74.74	6.82

**Figure 5:**
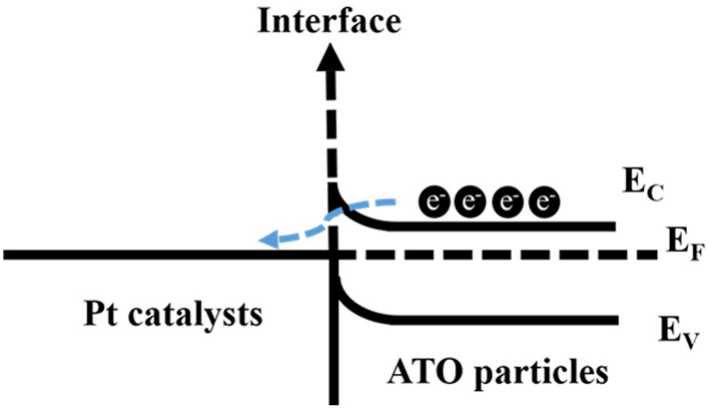
Schematic energy level diagrams for a Pt–ATO interface.

Figure 5 schematically illustrates the mechanism of charge transfer between Pt and ATO. A Schottky junction is created when metallic Pt comes into contact with semiconductor ATO, resulting in a barrier that inhibits the flow of electrons. Owing to the higher Fermi level of Pt compared with ATO, a built-in electric field is created. In this study, the interface barrier and defects between Pt and ATO is not high, allowing the electric field to drive electron flow from ATO to Pt. The ATO sample with a higher level of Sb doping provides more electrons available for transfer, thereby enhancing the charge transfer in the Pt–ATO junction. The modification of the charge density and electronic properties at the Pt surface in a Schottky junction has several beneficial effects. First, they reduce the adsorption of poisoning species, such as CO, on the surface of a Pt catalyst and thus help maintain the availability of active sites for desired reactions^42–44^. Second, the incorporated ATO enhances the occupancy of the Pt d-band, thereby weakening the Pt–O bond strength. Consequently, the weakened Pt–O bond promotes the cleavage of O–O bonds, facilitating the reaction with OH^−^ to form H_2_O at elevated potential and releasing Pt active sites^45,46^. Third, the weakening of Pt–O bond strength provides a favorable condition that promote increase in metallic Pt content. As the level of Sb doping increased, the ratio of metallic Pt showed a gradual increase from 43.6% in Pt/C to 64.8% in Pt/C–ATO 20%, as shown in Table 2. This finding serves as additional evidence for the robust metal–support interactions between Pt and ATO^37,47^. Finally, when ATO is incorporated into a system, it introduces additional oxygen vacancies and promotes the formation of hydroxyl groups (OH^−^) on the its surface. By enriching the presence of hydroxyl groups through ATO incorporation, the availability of surface oxygen species is enhanced, facilitating the reaction between poisoning CO intermediates absorbed on Pt sites and these oxygen species; this effect is typically attributed to the bifunctional mechanism^48^, the “ligand effect”^49^, or a synergistic combination of these factors^28^. The co-catalysis effect provided by the above-mentioned metal–semiconductor junction can enhance the overall catalytic activity of a Pt catalyst, facilitating efficient fuel utilization and improving the stability of fuel-cell systems.

The doping level of Sb in ATO was determined using XPS, revealing Sb doping amounts of 3.4%, 7.3%, and 13.5% for ATO-5%, ATO-10%, and ATO-20% particles, respectively. ATO-5%, ATO-10%, and ATO-20% particles exhibited electrical conductivity of 0.07, 0.21, and 0.13 S/cm, respectively, which were lower than the electrical conductivity of carbon black. When Sb is doped into SnO_2_, it takes on a donor role by substituting Sn and carrying a positive charge, resulting in Sb^5+^. Given the unique electronic structure of Sb^5+^, it donates additional electrons to the conduction band of a SnO_2_ structure. These extra electrons move within the conduction band, enhancing its electrical conductivity. As the level of Sb doping increases, the Sb^5+^ dopant gradually gives way to Sb^3+^, which acts as a compensating species for oxygen vacancies and consequently lowers electrical conductivity^50^. Moreover, the presence of excessive lattice defects, internal stress, and amorphous antimony oxide can contribute to decline in the electrical conductivity of ATO.

**Figure 6:**
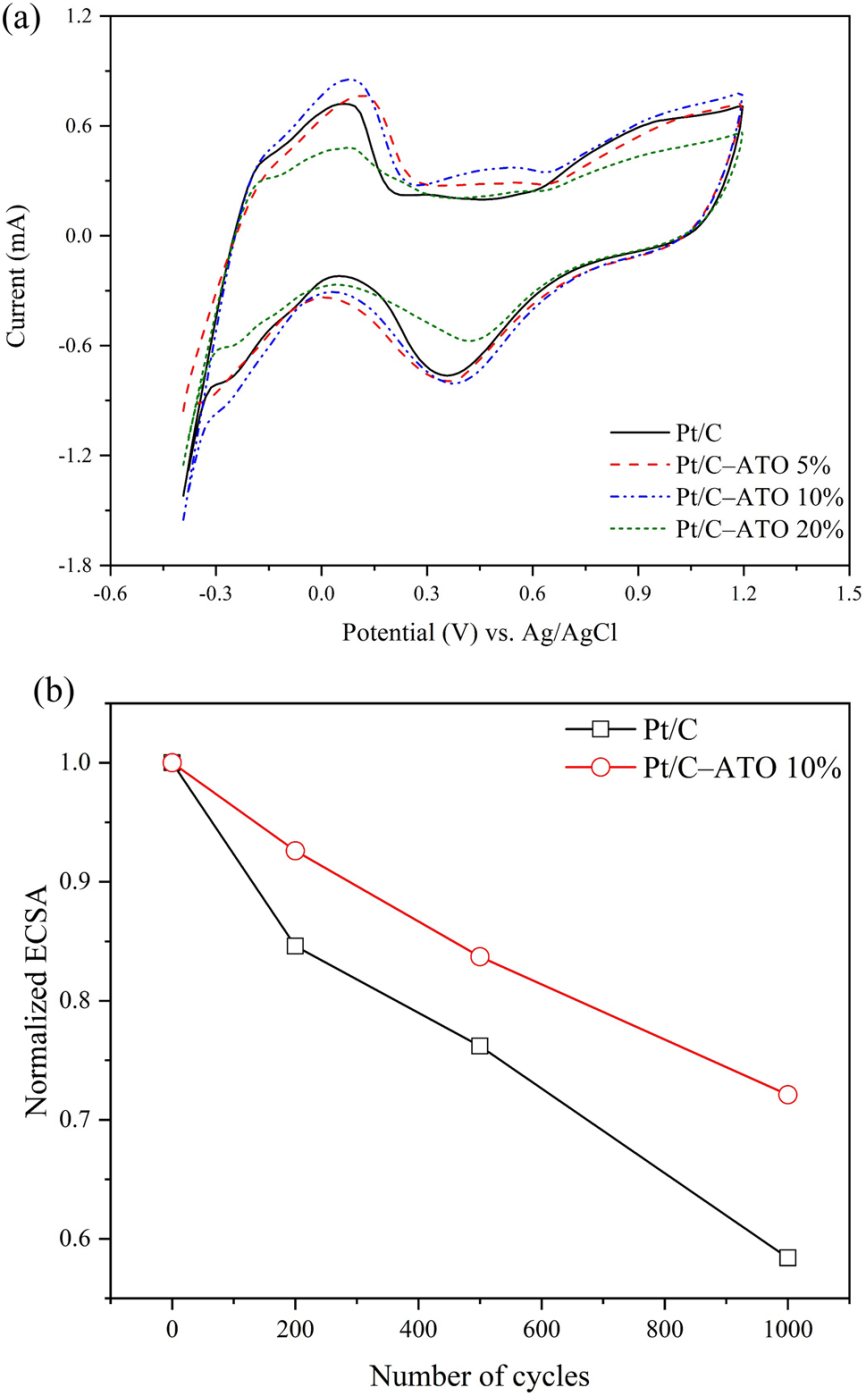
(**a**) Cyclic voltammogram of Pt/C and Pt/C–ATO catalysts in a 0.5 M H_2_SO_4_ solution. (**b**) Normalized ECSA of Pt/C and Pt/C–ATO 10% catalysts as a function of cycles.

Figure 6a shows the cyclic voltammograms of the Pt/C and Pt/C–ATO catalysts in a 0.5 M H_2_SO_4_ solution. The potential range from − 0.4 to 1.2 V exhibited distinct and well-defined hydrogen adsorption/desorption peaks. In a polycrystalline Pt electrode, multiple hydrogen absorption peaks are usually observed due to the presence of various crystal facets. However, if the electrode surface is predominantly composed of a specific crystal facet or exhibits a poorer crystallinity, there may be only one main hydrogen absorption peak^38,51^. The calculation of the ECSA of the Pt catalyst per unit weight of Pt was performed using the following equation.

$$ {\text{ECSA}} = \frac{{{\text{Q}}_{{\text{H}}} \left( {\upmu {\text{C}}/{\text{cm}}^{2} } \right)}}{{210\left( {\upmu {\text{C}}/{\text{cm}}^{2} } \right) \times {\text{W}}_{{{\text{Pt}}}} \left( {{\text{mg}}/{\text{cm}}^{2} } \right)}} $$

where Q_H_ is the charge of the absorption or desorption hydrogen on Pt surface, which corresponds to the area of cathode peak during the negative scan or anode peaks during the positive scan; W_Pt_ is the Pt loading by mass weight on the effective reaction area of the working electrode. ECSA is determined by extrapolation of the double-layer charging current from the region between 0.2 and 0.4 V back to about − 0.2 V and integrating the charge for oxidation of adsorbed hydrogen on a positive-going potential sweep. The ECSA values of Pt/C, Pt/C–ATO 5%, Pt/C–ATO 10%, and Pt/C–ATO 20% were 55.97, 55.45, 68.14, and 35.57 m^2^/g, respectively. The addition of ATO can improve the catalytic activity of Pt/C catalysts, increasing their efficiency in oxidation–reduction reactions. However, if the level of Sb doping is insufficient, ATO may exhibit poor conductivity, which decreases efficiency in oxidation–reduction reactions. When Sb doping exceeds the optimal level, it promotes the growth of the grain size of Pt, along with agglomeration and poor dispersion on the carbon support, resulting in a decrease in the utilization of Pt. In combination with the deteriorated electrical conductivity caused by excessive doping, a significant decrease in ECSA was observed. The Pt/C–ATO 10% catalyst achieved an optimal ECSA of 68.14 m^2^/g as a result of the well-balanced conductivity, active surface area, and metal–support interaction. Stability is a significant challenge in PEMFC technology. To assess the electrochemical stability of the prepared samples, CV with up to 1000 cycles was determined. Figure 6b illustrates loss in the relative ECSA of the Pt/C, Pt/C–ATO 5%, Pt/C–ATO 10%, and Pt/C–ATO 20% catalysts after 200, 500, and 1000 cycles. As the cycling progressed, a pronounced decrease in ECSA was observed for Pt/C, whereas Pt/C–ATO showed a gradual decline. In Pt/C, the total ECSA loss was approximately 42%, whereas in Pt/C–ATO, the loss was reduced to 28%. The findings suggested that using a combination of carbon black and ATO as a support provides results in electrochemical stability approximately 1.23 times that obtained when carbon black alone is used. This enhanced durability can be attributed to the superior corrosion resistance of ATO in an acidic environment, as well as the strong interaction between Pt and ATO.

**Figure 7:**
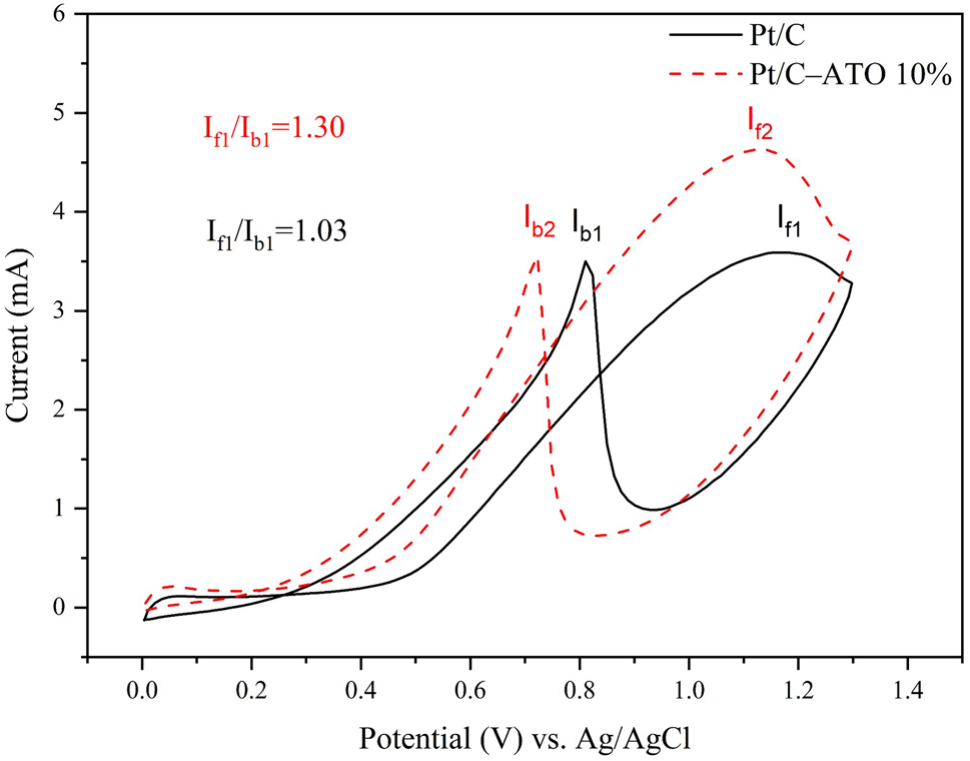
Cyclic voltammogram of Pt/C and Pt/C–ATO 10% catalysts in a 0.5 M H_2_SO_4_ + 1.0 M CH_3_OH solution.

To evaluate catalyst activity in methanol electrooxidation, steady-state polarization plots were obtained for Pt/C and Pt/C–ATO 10% catalysts in a solution containing 0.5 M H_2_SO_4_ and 1.0 M CH_3_OH, as shown in Fig. 7. The Pt/C–ATO 10% catalyst exhibits an onset oxidation potential of 0.413 V for MOR, and Pt/C catalyst has an onset oxidation potential of 0.478 V. This result suggested that MOR preferentially occurred on the Pt/C–ATO 10% catalyst. The peak current for MOR on the Pt/C–ATO 10% catalyst was 4.63 mA, which is 1.29 times the peak current observed on the Pt/C catalyst (3.59 mA), indicating the enhancing effect of ATO on the electrooxidation of methanol. The CO tolerance of Pt catalyst can be assessed by analyzing the current peak ratio of I_f_ (forward peak current density) to I_b_ (backward current peak density), represented as I_f_/I_b_^52^. Pt/C–ATO 10% exhibited a higher I_f_/I_b_ ratio of 1.30 compared to the I_f_/I_b_ ratio of 1.03 for Pt/C, indicating better efficiency in methanol oxidation to carbon dioxide and reduced accumulation of carbonaceous residues on the Pt surface. The results of onset oxidation potential, peak current, and I_f_/I_b_ ratio values demonstrated that the Pt/C–ATO 10% catalyst exhibited superior electrocatalytic activity and CO tolerance for MOR.

**Figure 8:**
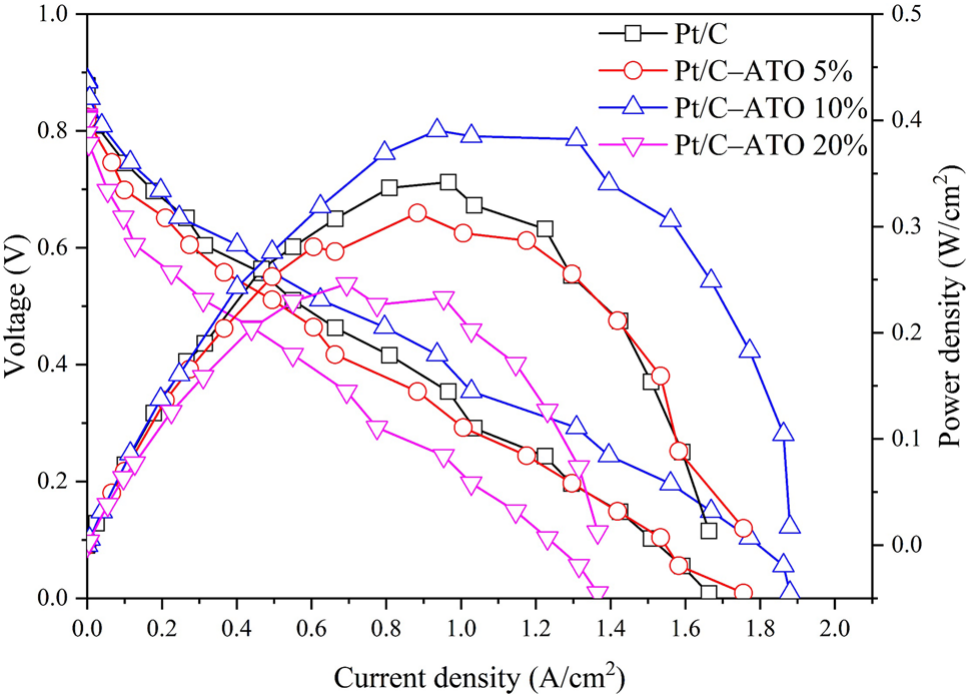
Polarization and power density curves of Pt/C and Pt/C–ATO catalysts.

The cell performance of Pt/C and Pt/C–ATO catalysts was examined under room temperature, which closely mimicked a nonhumidified state (RH = 0%), as shown in Fig. 8. The results directly verified the self-humidification capability of the MEA^53,54^. The current densities obtained at 0.6 V exhibited the following trend: Pt/C–ATO 10% > Pt/C > Pt/C–ATO 5% > Pt/C–ATO 20%. The Pt/C–ATO 10% catalyst exhibited a maximum power density of 0.39 W cm^−2^, surpassing the Pt/C catalyst (0.34 W cm^−2^) by 15%. These findings are consistent with the cyclic voltammograms measurements. Electrical conductivity, active surface area, and intrinsic activities are crucial factors that influence the cell performance of catalysts. Although the Pt/C–ATO 5% demonstrates a finer granularity of Pt particles, this advantage is offset by a lower electrical conductivity, resulting in virtually no change in electrochemical activity^51,55^. Despite the lower electrical conductivity and surface area of ATO compared with carbon black, the Pt/C–ATO 10% samples demonstrated higher surface specific activities. This result suggested that the higher intrinsic activities of Pt/C–ATO 10% outweighed the negative effect of the lower electrical conductivity and surface area of ATO, making the catalyst a promising catalyst support material in PEMFCs.

## Conclusion

In this work, Pt and ATO nanoparticles were simultaneously precipitated onto the surface of carbon black for the fabrication of a highly effective catalyst for PEMFCs. The influences of Sb doping on the crystal, structural, electrochemical characteristics of the ATO particles were examined. Through the proper incorporation of Sb dopants, ATO exhibited a remarkable increase in electrical conductivity while maintaining uniform dispersion on the carbon support without evident agglomeration or coalescence. The C–ATO-supported Pt nanoparticles exhibited a negative shift in the binding energy of Pt 4f and a higher ratio of metallic Pt owing to the formation of unique Pt/C–ATO triple-junction nanostructures and strong interaction with ATO. Accordingly, the Pt/C–ATO catalysts exhibited significantly enhanced electrochemical activity, durability, and CO tolerance compared with the Pt/C catalyst according to the CV measurements. The MEA samples containing a composite support of carbon and ATO in the catalyst layer exhibited a power density up to 15% higher than that of the MEA lacking ATO particles, indicating that achieving optimal cell performance relies on finding the right balance among electrical conductivity, active surface area, and intrinsic activity.

## Data Availability

The datasets generated during and/or analysed during the current study are available from the corresponding author on reasonable request.
